# Changes in health care utilization and financial protection after integration of the rural and urban social health insurance schemes in Beijing, China

**DOI:** 10.1186/s12913-022-08602-1

**Published:** 2022-10-03

**Authors:** Zhenyu Shi, Ping He, Dawei Zhu, Feng Lu, Qingyue Meng

**Affiliations:** 1 Department of Health Policy and Management, School of Public Health, Peking University, Beijing, China; 2 China Center for Health Development Studies, Peking University, Beijing, China; 3 Beijing Municipal Health Big Data and Policy Research Center, Beijing, China

**Keywords:** Health equity, Health insurance, Rural health services, Reimbursement, Risk sharing

## Abstract

**Background:**

China expanded health coverage to residents in informal economic sectors by the rural new cooperative medical scheme (NCMS) for rural population and urban resident basic medical insurance scheme (URBMI) for non-working urban residents. Fragmentation of resident social health insurance schemes exacerbated the health inequity and China started the integration of urban and rural resident medical insurance schemes since 2016. Beijing finished the insurance integration in 2017 and has been implementing a unified urban and rural resident basic medical insurance scheme (URRBMI) since the beginning of 2018. This study aims to examine changes in health care utilization and financial protection after integration of the rural and urban social health insurance schemes.

**Methods:**

We used household survey data from Beijing Health Services Survey in 2013 and 2018. Respondents who were 15 or older and covered by URBMI, NCMS or URRBMI were included in this study. Our study finally included 8,554 individuals in 2013 and 6,973 individuals in 2018, about 70% of which were rural residents in each year. Descriptive analysis was used to compare the healthcare utilization, healthcare expenditure and incidence of catastrophic health expenditure between different groups. A series of two-part regression models were used to analyze the changes of healthcare utilization, healthcare expenditure and incidence of catastrophic health expenditure.

**Results:**

From 2013 to 2018, urban–rural disparity in outpatient care utilization seemed widened because urban residents’ utilization of outpatient care increased 131% while rural residents’ utilization only increased 72%; both rural and urban residents’ spending on outpatient care increased about 50%. Utilization of inpatient care changed little and poor residents still used significantly less inpatient care compared with the rich residents. Poor residents still suffered heavily catastrophic health expenditures.

**Conclusion:**

From 2013 to 2018, residents’ utilization of healthcare, especially outpatient care, increased in Beijing. Health insurance reforms increased residents’ utilization of healthcare but failed to reduce their healthcare financial burden, especially for poor people. Our study advocates more pro-poor insurance policies and more efforts on the efficiency of health system.

**Supplementary Information:**

The online version contains supplementary material available at 10.1186/s12913-022-08602-1.

## Background

How to expand health insurance coverage to the informal economic sectors is a worldwide concern. Facing this challenge, China started to introduce the rural new cooperative medical scheme (NCMS) for rural population and urban resident basic medical insurance scheme (URBMI) for non-working urban residents in 2003 and 2007, respectively. About 70% of the funds were from the government subsidy for the both schemes [1]. China’s NCMS and URBMI expanded rapidly. By 2015, the both schemes covered almost all the target population with 670 million rural people and 377 million urban people covered. From 2007 to 2015, per capita fund for NCMS increased from 60 RMB yuan to 490 RMB yuan; for URBMI increased from 100 RMB yuan to 560 RMB yuan [2, 3]. The NCMS and URBMI, as well as the urban employee basic medical insurance (UEBMI) for formal workers established in 1997, constitute the main part of health security system in China and cover more than 95% of the population. Generally speaking, UEBMI provided the most generous benefit packages for the insured and NCMS provided the least generous benefit packages.

Studies found that the expansion of social health insurance improved access to healthcare of the insured but had limited or negative impact on the financial protection [4–6]. Wagstaff et al. [4] and Hou et al. [5] found that NCMS increased the probability of using outpatient and inpatient care. Liu and Zhao [6] found that URBMI increased utilization of medical services. As for financial protection, most studies found that China’s basic medical insurance didn’t reduce the out-of-pocket health expenditure [4–7]. Since financial burden is not only associated with the out-of-pocket health expenditure but also associated with residents’ capacity to pay, most studies used catastrophic health expenditure (CHE) as the indicator for financial protection [8–13]. Li et al. [9] found that the poorest residents spent the least but their incidence of CHE was the highest. Households covered by UEBMI and URBMI had lower risk of CHE than those without insurance. Xie et al. [14] found that NCMS significantly decreased residents’ CHE. Song et al. [15] found that the upward trends in the incidence of CHE ceased after 2002 when China start the insurance coverage for non-working residents. Zhou et al. [16] found that having NCMS was associated with a lower incidence of catastrophic health expenditure (CHE). Li et al. [17] conducted a systematic review and found that the basic health insurance plan has reduce the rate of CHE.

For countries with more than one health insurance schemes, the equity of health insurance system is always of great concern. Integration of social health insurance schemes or cross-subsidies between different people are critical strategies to improve health equity. Thailand, Korea and Turkey have demonstrated process of integrating their health insurance schemes [1, 18–21]. Japan and Germany have tried to unify the coinsurance and health care provision for all people [22–24]. In China, to improve equity in health has been an aim to be achieved during the health system reform over the past decade [25]. One of the strategies to reduce disparities in health financing and care of the people is to integrate the NCMS and URBMI [1, 26]. China began to integrate the rural and urban insurance schemes in 2016, aiming to form a unified insurance scheme called urban and rural resident basic medical insurance scheme (URRBMI) at municipal level by the end of 2019, to equalize the benefit packages for both rural and urban residents. By May, 2019, there were 24 provinces finishing the insurance integration [27].

Few studies were found on health insurance integration in China and the evidence about effects of the integration on health care utilization and financial protection was mixed. While some studies found that insurance integration increased middle-aged and elderly people’s probability of inpatient care utilization or their frequency of healthcare utilization [28, 29], some studies did not report that the integration has increased health care utilization for the rural people [30]. Similarly, there was no consistent finding on effect of the integration on financial protection for the rural people [30, 31].

Beijing as the capital city of China has been highly urbanized, in 2020, in total of 21.9 million population in Beijing, 12.5% of which was rural people, with 23.7 percentage lower of rural population proportion than the country average [32]. Gaps of social protection programs between rural and urban people exist in Beijing, and the integration of NCMS and URBMI aim to mitigate these gaps.

Before the integration, there were many differences between URBMI and NCMS in Beijing. Firstly, URBMI was financed at municipal level and NCMS was financed at district level, which meant Beijing had 13 NCMS schemes (in 13 districts) and 1 URBMI schemes. Secondly, for the insured of URBMI in any district, per capita fund was unified, but per capita fund could be different for the insured of NCMS in different districts. For example, the per capita fund was 1560 RMB yuan for the insured of NCMS in Haidian district but only 1200 RMB yuan for the insured of NCMS in Huairou district. Thirdly, all the insured with URBMI enjoyed a same benefit package but rural residents in different NCMS schemes might have very different benefit package. Reimbursement rate in Beijing were significantly different between URBMI and NCMS or between different NCMSs. For instance, URBMI reimbursed 70% of the covered inpatient spending in tertiary hospitals, NCMS in Shunyi district reimbursed 55%-60%, and NCMS in Huairou district reimbursed 50%. In addition, URBMI and NCMS covered different hospitals. NCMS usually only covered hospitals within districts and if the insured of NCMS was hospitalized in hospitals in other districts, the reimbursement rate would be significant low or even as low as 0.

Beijing started the implementation of URRBMI in the January 1^st^, 2018. After integration, both rural and urban non-working residents (all the insured of URBMI and NCMS before 2018) are eligible for the URRBMI scheme which is financed at municipal level. The insured of URRBMI in any district have a unified per capita fund and enjoy the same benefit package. The fund pool was significantly expanded by integrating the resident-based health insurance schemes and it was expected that the rural people could benefit more from the integration due to the increasing power of risk sharing. URRBMI covered all the hospitals that previously covered by URBMI and NCMS thus the insured of URRBMI have more choices when they need health care.

Using the opportunity of integrating social health insurance schemes China, this study aims to examine changes in health care utilization and financial protection after integration of the rural and urban social health insurance schemes. Our study might contribute to previous literature in following aspects. Firstly, Beijing is one of the few big municipal cities in China that can demonstrate the best situation of efforts in improving equity in health. Our study showed that even in Beijing’s health system, equity of healthcare utilization and financial protection between residents with different socioeconomic status could not be achieved merely by insurance integration. Secondly, there are many differences on health systems between regions in China. Compared with many studies using nationwide survey data, we focused on Beijing’s insurance integration. Based on the health system in Beijing, our study was able to further explored the possible mechanism behind the changes of healthcare utilization and financial protection after insurance integration.

## Methods

### Data

China conducts National Health Service Surveys (NHSS) every 5 years to regularly collect information on people’s health status, health service utilization, medical spending and so on. Beijing Health Service Surveys (BHSS) is part of the NHSS. Data in this study was collected from two rounds of BHSS in 2013 and 2018. Since the integrated insurance scheme took effect since the start of 2018 and BHSS 2018 was conducted at the end of the third quarter, we expected to observe the initial effect of the insurance integration in Beijing.

BHSS selected the sample with a multistage cluster sampling method. 10 districts in 2013 and 16 districts in 2018, five towns or communities from each district, two villages or neighborhoods from each town or community and 60 households in each village or neighborhood were randomly selected as the sample of BHSS. Additionally, BHSS expanded several districts’ sample (3 districts in 2013 and 5 districts in 2018) because of their population were significantly larger than others. Well-trained local medical workers were the main interviewers and instructors of BHSS. Interviewers and instructors were responsible for data collection and quality assurance, respectively. Finally, BHSS surveyed 20376 residents (7270 rural residents) in 2013 and 29196 residents (10895 rural residents) in 2018.

BHSS asked respondents’ insurance status in each wave. Since respondents with UEBMI were not the policy target of insurance integration, only respondents who were 15 or older and covered by URBMI, NCMS or URRBMI were included in this study. Since NCMS schemes were different between districts in Beijing and only 10 districts were surveyed in 2013, we only included residents of these 10 districts in each wave to ensure respondents in 2013 and 2018 comparative. Following Wagstaff and Lindelow’s handling of outliers [4], individuals with top 1% annual out-of-pocket health expenditure in each wave was been trimmed. We also trimmed individuals with the bottom 1% per capita household consumption in each wave because several households reported consumption as low as near zero. In addition, we droped the observation if respondents’ health expenditure exceeds his/her household’s total consumption. Our final sample included 8,554 individuals in 2013 and 6,973 individuals in 2018, about 70% of which were rural residents in each year.

### Variable

#### Dependent variables

The outcomes of interest in our study were healthcare utilization, health spending and financial protection. For healthcare utilization, we constructed two binary variables: whether respondents use any outpatient care in the past two weeks and whether respondents use any inpatient care in the past 12 months. For health spending, we calculated respondents’ annual out-of-pocket spending on hospitalization and their two-week out-of-pocket spending on outpatient care, respectively.

In our study, we constructed a binary variable at individual level to identify whether respondents had CHE. Following previous research [4, 33], people who annually spent 25% or more of its annual per capita household non-food consumption on health services were defined as suffering CHE in our study. The formula of CHE was shown as follows:

$$E_i=1\;\mathrm{if}\;\frac{OOP_i}{PCHNFC_i}\geq0.25$$

$$E_i=0\;\mathrm{if}\;\frac{OOP_i}{PCHNFC_i}<0.25$$

$$CHE=\frac{1}{i}\sum_{i=1}^{i}{E}_{i}$$

${E}_{i}$ is measuring whether individual $i$ suffered CHE, $OO{P}_{i}$ is the sum of annual spending on outpatient care, inpatient care and self-treatment of individual $i$, $PCHNF{C}_{i}$ is the per capita household non-food consumption of individual $i$, $CHE$ is the incidence of CHE. Considered that BHSS only investigated respondents’ two-week spending on outpatient care and self-treatment, we respectively multiplied the spending by 26 to estimate the annual spending on outpatient care and self-treatment. We also constructed a household level CHE and our results were robust. The results of household level CHE were shown in supplementary file.

#### Independent variables

In this study, we defined different socioeconomic groups by hukou (rural or urban) and living standards. BHSS asked whether the respondent was rural or urban residents. We constructed variable *hukou* and its value is 1 for rural residents and 0 for urban residents. Before insurance integration, rural residents ineligible for UEBMI were covered by NCMS and urban residents ineligible for UEBMI were covered by URBMI. Therefore, in our study, the urban–rural disparity in outcome variables also measured the disparity between residents with different insurance schemes.

We measured living standards by per capita annual household consumption (PCHC), which was perceived as a better indicator than income especially for developing countries [34]. In each wave of BHSS, we constructed a variable *PCHC* to indicate the relevant living standards of the respondents. For rural residents in BHSS 2013, we ranked their per capita annual household consumption; divided them into 5 groups by the rank and constructed a categorical variable *PCHC* (1–5, 1 means the poorest group) to indicate which categories a respondent belonged to. For urban residents in BHSS 2013, we repeated this process and also divided them into 5 groups. Thus, it’s possible that rural residents with *PCHC* valued 2 might be worse off than the urban residents with *PCHC* valued 1. For rural and urban residents in BHSS 2018, the same process was done to constructed the variable *PCHC* for them, just as we did for residents in BHSS 2013.

There were also a series of covariates in our model. Referring to previous study [9], covariates at individual level of our study included age, the square of age, gender, marital status, occupation status, education level, self-assessed health status, whether having any chronic disease and whether having a private health insurance. Since we constructed CHE at individual level, we could control each household members’ characteristics instead of the characteristics of the head of household. Covariates at household level included household size. Age related variables were continuous; variables of gender, marital status, occupation status and self-assessed health status, chronic diseases and private health insurance were binary variables. For self-assessed health status, respondents with an 80 or higher self-reported health status score were defined healthy (for both respondents in 2013 and 2018, their median self-reported health status score was 80). Education level and household size were categorical variables. Respondents were classified into 3 groups by education level: primary school or below, junior high school and senior high school or above.

### Analytic methods

#### Descriptive methods 

In descriptive analysis, we firstly compared healthcare utilization of different socioeconomic residents. Secondly, we respectively compared healthcare users’ two-week spending on outpatient care and 12-month spending on inpatient care. Then, we compared the incidence of CHE of different socioeconomic residents. For binary variables, we tested the difference between residents with χ^2^ test. For expenditure variables, we tested the difference of their logarithms with ANOVA.

#### Model estimation

Given that there were many observations with zero health expenditure, hurdle model (or two-part model) was used with a logistic regression in the first part and log-linear regression in the second part [35, 36]. The logistic part estimated the probability of using certain healthcare (healthcare utilization) and the log-linear part estimated the spending on certain healthcare for healthcare users (healthcare spending). For financial protection, we just used the logistic part of the model to estimate residents’ incidence of CHE.

We developed Model 1 to analyze disparities in our outcome variables between rural and urban residents. Model 1 was constructed as follows:

Model 1, logistic part:

$$\begin{array}{cc}logit\left(P\right)&=\alpha_0+\sum\limits_{q=2}^5\gamma_q\left(PCHC=q\right)+\lambda_jX_j+\delta_d+\epsilon\\&+\alpha_1rural+\sum\limits_{h=0}^1\beta_h\left(rural=h\right)\ast\left(year=2018\right)\end{array}$$

Model 1, log-linear part:

$$\begin{array}{cc}ln\left(y|y>0\right)& ={\alpha }_{0}^{\mathrm{^{\prime}}}+\sum\limits_{q=2}^{5}{\gamma }_{q}^{\mathrm{^{\prime}}}\left(PCHC=q\right)+{\lambda }_{j}^{\mathrm{^{\prime}}}{X}_{j}+{\delta }_{d}^{\mathrm{^{\prime}}}+{\epsilon }^{\mathrm{^{\prime}}}\\ & +{\alpha }_{1}^{\mathrm{^{\prime}}}rural+\sum\limits_{h=0}^{1}{\beta }_{h}^{\mathrm{^{\prime}}}\left(rural=h\right)*\left(year=2018\right)\end{array}$$

${\alpha }_{1} \left({{\alpha }^{\mathrm{^{\prime}}}}_{1}\right)$ captured the average differences on outcome variables between rural and urban residents in 2013. ${\beta }_{h} ({\beta \mathrm{^{\prime}}}_{h})$ captured the average changes on outcome variables between 2013 to 2018 for urban residents (h = 0) and rural residents (h = 1), respectively. In the case of hospitalization, for example, ${\alpha }_{1}$ meant that, in 2013, the utilization of hospitalization for rural people was averagely $\mathrm{exp}\left({\alpha }_{1}\right)$ times of that for urban people. ${\beta }_{0}$ meant that, in 2018, the utilization of hospitalization for urban residents increased to ${\mathrm{exp}(\beta }_{0})$ times of that in 2013. Similarly, ${\beta }_{1}$ captured the this change for the rural. In the second part of model 1, ${{\alpha }^{\mathrm{^{\prime}}}}_{1}$ meant that, in 2013, the annual spending on hospitalization for rural hospitalized people was averagely $\mathrm{exp}\left({\mathrm{\alpha }}_{1}^{^{\prime}}\right)$ times of that for urban people. ${\beta }_{0}^{^{\prime}}$ meant that, in 2018, the annual spending on hospitalization for urban hospitalized people increased to $\mathrm{exp}\left({\beta }_{0}^{^{\prime}}\right)$ times of that in 2013. Similarly, ${\beta }_{1}^{^{\prime}}$ captured the this change for the rural.

Model 2 was designed to analyze disparities in our outcome variables between residents with different household consumption. Model 2 was constructed as follows:

Model 2, logistic part:

$$\begin{array}{cc}logit\left(P\right)& ={\alpha }_{0}+{\alpha }_{1}rural+{\lambda }_{j}{X}_{j}+{\delta }_{d}+\epsilon \\ & +\sum\limits_{q=2}^{5}{\gamma }_{q}\left(PCHC=q\right)+\sum\limits_{q=1}^{5}{\theta }_{q}\left(PCHC=q\right)*\left(year=2018\right)\end{array}$$

Model 2, log-linear part:

$$\begin{array}{cc}ln\left(y|y>0\right)& ={\alpha }_{0}^{\mathrm{^{\prime}}}+{\alpha }_{1}^{\mathrm{^{\prime}}}rural+{\lambda }_{j}^{\mathrm{^{\prime}}}{X}_{j}+{\delta }_{d}^{\mathrm{^{\prime}}}+{\epsilon }^{\mathrm{^{\prime}}}\\ & +\sum\limits_{q=2}^{5}{\gamma }_{q}^{\mathrm{^{\prime}}}\left(PCHC=q\right)+\sum\limits_{q=1}^{5}{\theta }_{q}^{\mathrm{^{\prime}}}\left(PCHC=q\right)*\left(year=2018\right)\end{array}$$

${\gamma }_{q} ({\gamma \mathrm{^{\prime}}}_{q})$ captured the difference in certain outcome variable between residents in the q^th^ economic group and the poorest group of residents. ${\theta }_{q} ({\theta \mathrm{^{\prime}}}_{q})$ captured changes on certain outcome variable for the q^th^ economic group between 2013 to 2018.

Model 3 was developed to analyze disparities in outcome variables between different socioeconomic residents (hukou and PCHC). Model 3 was constructed as follows:

Model 3, logistic part:

$$\begin{array}{cc}logit\left(P\right)& ={\alpha }_{0}+\sum\limits_{q=2}^{5}{\gamma }_{q}\left(PCHC=q\right)+{\lambda }_{j}{X}_{j}+{\delta }_{d}+\epsilon \\ & +\sum\limits_{q=1}^{5}{\kappa }_{q}\left(PCHC=q\right)*\left(rural=1\right)+\sum\limits_{q=1}^{5}\sum\limits_{h=0}^{1}{\omega }_{qh}\left(PCHC=q\right)*\left(rural=h\right)*\left(year=2018\right)\end{array}$$

Model 3, log-linear part:

$$\begin{array}{cc}ln\left(y|y>0\right)& ={\alpha }_{0}^{\mathrm{^{\prime}}}+\sum\limits_{q=2}^{5}{\gamma }_{q}^{\mathrm{^{\prime}}}\left(PCHC=q\right)+{\lambda }_{j}^{\mathrm{^{\prime}}}{X}_{j}+{\delta }_{d}^{\mathrm{^{\prime}}}+{\epsilon }^{\mathrm{^{\prime}}}\\ & +\sum\limits_{q=1}^{5}{\kappa }_{q}^{\mathrm{^{\prime}}}\left(PCHC=q\right)*\left(rural=1\right)+\sum\limits_{q=1}^{5}\sum\limits_{h=0}^{1}{\omega }_{qh}^{\mathrm{^{\prime}}}\left(PCHC=q\right)*\left(rural=h\right)*\left(year=2018\right)\end{array}$$

${\kappa }_{q} ({\kappa \mathrm{^{\prime}}}_{q})$ captured the urban–rural difference on certain outcome variable within the q^th^ economic group in 2013 (urban residents as the reference in each group). ${\omega }_{qh} ({\omega \mathrm{^{\prime}}}_{qh})$ captured changes on certain outcome variable between 2013 to 2018 for urban residents (h = 0) or rural residents (h = 1) in the q^th^ economic group.

**Fig. 1:**
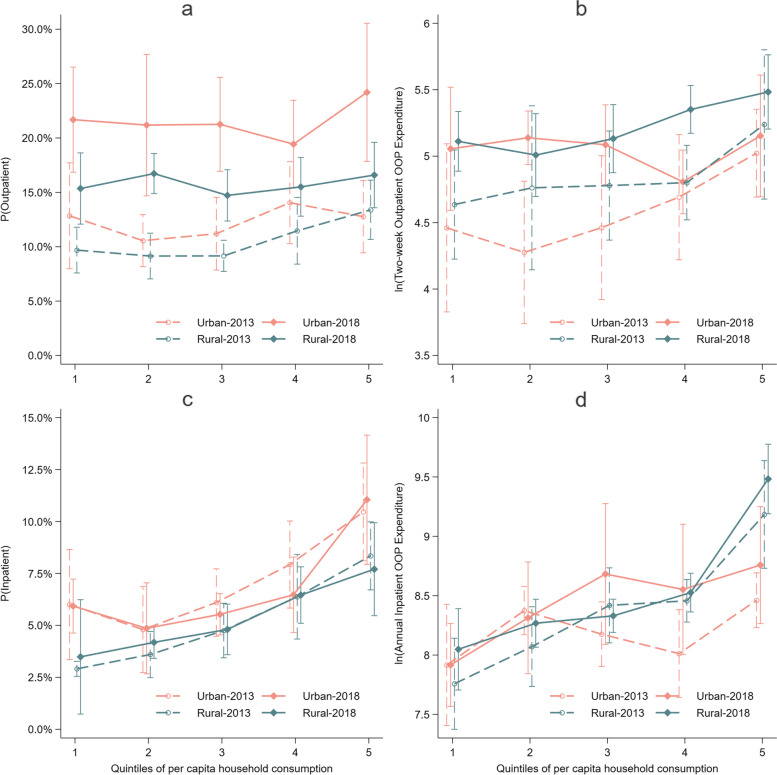
Healthcare utilization, expenditure for residents with different household consumption, by hukou, 2013 and 2018

**Fig. 2:**
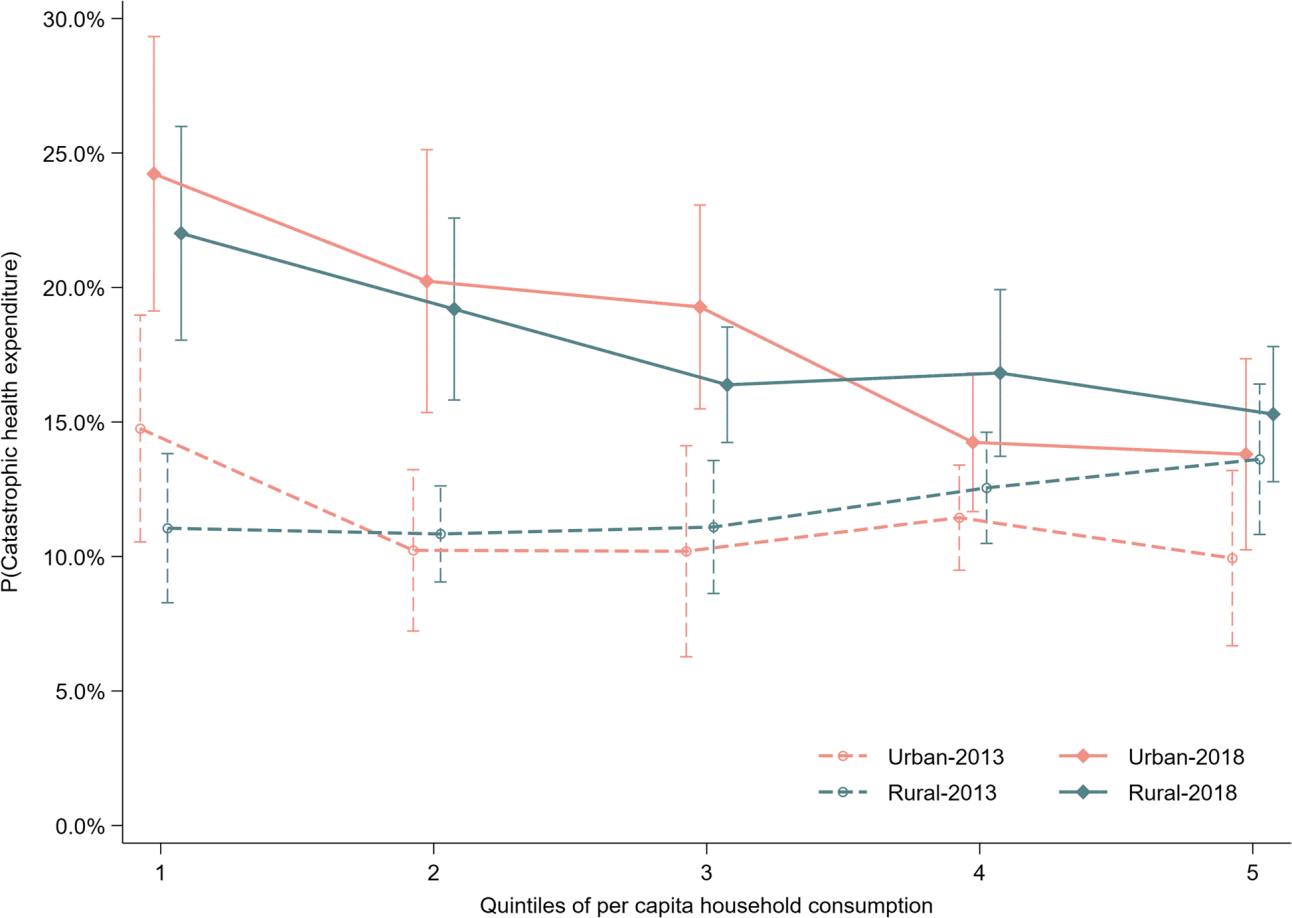
Incidence of catastrophic health expenditure for residents with different household consumption, by hukou, 2013 and 2018

To visualize our results, regression results on healthcare utilization and healthcare spending of different socioeconomic residents were shown in Fig. 1; regression results on their incidences of CHE were shown in Fig. 2.

## Results

As shown in Table 1, in 2013, rural residents were less likely to use both outpatient and inpatient care. But rural residents spent more once they use healthcare. The urban–rural disparities in utilization and expenditure of inpatient care became insignificant in 2018. For residents with different household consumption, both healthcare utilization and healthcare expenditure of the better off were higher in 2013; only disparities in outpatient care utilization became insignificant in 2018. Residents in different socioeconomic groups experience similar risk of suffering CHE in 2013 but the poorer residents bore relatively higher risk in 2018.

**Table 1:** Healthcare utilization, expenditure and catastrophic health expenditure, 2013 and 2018

	**Utilization (%) **^**a**^		**Expenditure (RMB yuan) **^**b**^		**CHE (%) **^**c**^
	**Outpatient**	**Inpatient**	**Outpatient**	**Inpatient**	
**2013**					
**By hukou**					
Urban	12.66	6.67	358.82	6835.28	11.23
Rural	9.16	4.64	432.38	9689.33	10.54
*p-value*	< *0.001*	< *0.001*	*0.0202*	*0.0061*	*0.3480*
**By household consumption**					
Q1 (poorest)	8.87	3.88	261.47	3772.96	10.72
Q2	8.13	3.77	312.02	4727.07	9.31
Q3	9.18	4.91	354.64	6500.24	10.34
Q4	11.70	6.11	333.85	6007.33	11.58
Q5 (richest)	13.10	7.52	662.43	16,634.61	11.75
*p-value*	< *0.001*	< *0.001*	< *0.001*	< *0.001*	*0.1350*
**2018**					
**By hukou**					
Urban	22.91	6.50	456.24	8925.09	18.65
Rural	17.55	5.82	553.03	8933.91	20.24
*p-value*	< *0.001*	*0.2690*	*0.0096*	*0.3052*	*0.1270*
**By household consumption**					
Q1 (poorest)	18.21	4.85	381.10	4778.99	24.40
Q2	20.43	5.00	440.74	5638.70	21.96
Q3	19.58	5.68	578.82	6694.59	19.79
Q4	18.55	6.78	524.09	7956.73	17.79
Q5 (richest)	19.06	7.92	675.58	16,603.88	14.53
*p-value*	*0.5940*	*0.0030*	*0.0451*	< *0.001*	< *0.001*

Binary variables are tested with χ2 test, expenditure variables’ logarithms are tested with ANOVA

^a^ Utilization measures the proportion of residents who used outpatient care in the past 2 weeks or who used inpatient care in the past 12 months

^b^ Expenditure measures outpatients’ two-week expenditure on outpatient care or inpatients’ annual expenditure on hospitalization. All expenditure results are converted to comparable expenditure in 2018 with Beijing’s CPI

^c^ *CHE* Catastrophic health expenditure

Table 2 shows that, from 2013 to 2018, the urban–rural disparity in outpatient care utilization seemed widened because urban residents’ utilization of outpatient care increased 131% while rural residents’ utilization only increased 72%; both rural and urban residents’ spending on outpatient care increased about 50% (Model 1). Coefficients in Model 2 shows that both poorer residents’ utilization and expenditure were lower in 2013 but increased more between 2013 to 2018. As a result, a narrowed gap was observed between residents with different household consumption, which was consistent with results in Table 1 and Fig. 1. Model 3 and Fig. 1 suggested the narrowed disparity in outpatient care utilization between residents with different PCHC was mainly due to the change within rural residents, but the narrowed disparity in spending was mainly due to the change within urban residents.

**Table 2:** Regression results for two-week outpatient care utilization and expenditure

	**Model 1**		**Model 2**		**Model 3**	
	**Part I**^**a**^	**Part II**^**b**^	**Part I**^**a**^	**Part II**^**b**^	**Part I**^**a**^	**Part II**^**b**^
**Rural**	0.83	1.31	0.71**	1.23**		
**PCHC (poorest as the reference)**						
Q2	0.99	0.98	0.87	1.01	0.77	0.83
Q3	0.93	1.05	0.89	1.09	0.83	1.00
Q4	1.05	1.13	1.19*	1.20	1.13	1.26
Q5 (richest)	1.22**	1.49**	1.31	1.79*	0.99	1.75
**Hukou#Year**						
Urban-2018	2.31***	1.59*				
Rural-2018	1.72***	1.45*				
**Year#PCHC**						
2018-Q1 (poorest)			1.95***	1.68**		
2018-Q2			2.41***	1.60*		
2018-Q3			2.08***	1.58**		
2018-Q4			1.55***	1.51***		
2018-Q5 (richest)			1.72**	1.22		
**Hukou#PCHC**						
Rural-Q1 (poorest)					0.69	1.19
Rural-Q2					0.83	1.63
Rural-Q3					0.78	1.37
Rural-Q4					0.76	1.12
Rural-Q5 (richest)					1.07	1.24
**Hukou#Year#PCHC**						
Urban-2018-Q1 (poorest)					2.18***	1.81
Urban-2018-Q2					2.72***	2.37***
Urban-2018-Q3					2.54***	1.87*
Urban-2018-Q4					1.62*	1.12
Urban-2018-Q5 (richest)					2.66***	1.14
Rural-2018-Q1 (poorest)					1.85**	1.61**
Rural-2018-Q2					2.25***	1.28
Rural-2018-Q3					1.87***	1.42
Rural-2018-Q4					1.51***	1.73***
Rural-2018-Q5 (richest)					1.36	1.28
**Observations**	15,489	2,206	15,489	2,206	15,489	2,206

All models included individual and household level covariates and district fixed effect. Standard errors adjusted for clustering at district level. All expenditure results were converted to comparable expenditure in 2018 with Beijing’s CPI. ***, **, and * indicated the significance at 1%, 5%, and 10% level, respectively

*PCHC* Per capita annual household consumption

^a^ For Part I, odds ratio was reported

^b^ For Part II, $\exp(\widehat\beta)$ was reported

As presented in Table 3, regressions for inpatient care show that, in 2013, rural residents were significantly less likely to use inpatient care than the urban. Rural residents spent more once they were hospitalized (not statistically significant). The urban–rural disparity in utilization rarely changed but the urban–rural disparity in spending seemed narrowed because urban residents’ expenditure increased 34% and that for the rural increased 16% (not statistically significant). However, results from model 3 implies that only richer urban residents’ expenditure increased. For both the utilization and expenditure of hospitalization, coefficients of model 2 depict obvious differences between poor and rich residents. What’s worse, the poorest rural residents’ utilization of inpatient care was only half of the poorest urban residents (Model 3). The utilization and expenditure of hospitalization for the poorest two groups of rural residents increased (not statistically significant), but the urban–rural disparity in utilization still remain in the poorest group (Fig. 1).

**Table 3:** Regression results for annual inpatient care utilization and expenditure

	**Model 1**		**Model 2**		**Model 3**	
	**Part I**^**a**^	**Part II**^**b**^	**Part I**^**a**^	**Part II**^**b**^	**Part I**^**a**^	**Part II**^**b**^
**Rural**	0.70***	1.33	0.73**	1.26		
**PCHC (poorest as the reference)**						
Q2	1.05	1.34**	1.04	1.39**	0.78	1.58*
Q3	1.30	1.57***	1.38*	1.61***	1.02	1.30
Q4	1.77***	1.60***	1.90***	1.56**	1.37	1.10
Q5 (richest)	2.44***	3.06***	2.60***	2.88***	1.90***	1.72**
**Hukou#Year**						
Urban-2018	0.95	1.34				
Rural-2018	1.04	1.16				
**Year#PCHC**						
2018-Q1 (poorest)			1.10	1.20		
2018-Q2			1.12	1.11		
2018-Q3			0.98	1.12		
2018-Q4			0.95	1.25*		
2018-Q5 (richest)			0.96	1.35		
**Hukou#PCHC**						
Rural-Q1 (poorest)					0.46***	0.85
Rural-Q2					0.73	0.74
Rural-Q3					0.76	1.27
Rural-Q4					0.78	1.56**
Rural-Q5 (richest)					0.77**	2.06**
**Hukou#Year#PCHC**						
Urban-2018-Q1 (poorest)					0.99	1.00
Urban-2018-Q2					1.01	0.94
Urban-2018-Q3					0.90	1.66*
Urban-2018-Q4					0.79	1.72
Urban-2018-Q5 (richest)					1.07	1.34
Rural-2018-Q1 (poorest)					1.21	1.34
Rural-2018-Q2					1.18	1.22
Rural-2018-Q3					1.01	0.92
Rural-2018-Q4					1.01	1.07
Rural-2018-Q5 (richest)					0.91	1.35
**Observations**	15,489	868	15,489	868	15,489	868

All models included individual and household level covariates and district fixed effect. Standard errors adjusted for clustering at district level. All expenditure results were converted to comparable expenditure in 2018 with Beijing’s CPI. ***, **, and * indicated the significance at 1%, 5%, and 10% level, respectively

*PCHC* Per capita annual household consumption

^a^ For Part I, odds ratio was reported

^b^ For Part II, $\exp(\widehat\beta)$ was reported

As shown in Table 4, incidence of CHE substantially increased in 2018. Disparities in CHE incidence between poor and rich group might widened because poor residents’ incidence of CHE grew at a higher growth rate. This trend in growth of CHE incidence, as depicted in model 3, was also applicable within urban residents and within rural residents.

**Table 4:** Regression results for incidence of catastrophic health expenditure

	**Model 1**	**Model 2**	**Model 3**
**Rural**	1.08	1.01	
**PCHC (poorest as the reference)**			
Q2	0.81***	0.84	0.62***
Q3	0.73***	0.85	0.62*
Q4	0.74***	1.00	0.72
Q5 (richest)	0.72***	1.02	0.60*
**Hukou#Year**			
Urban-2018	1.96***		
Rural-2018	1.78***		
**Year#PCHC**			
2018-Q1 (poorest)		2.42***	
2018-Q2		2.29***	
2018-Q3		1.88***	
2018-Q4		1.46***	
2018-Q5 (richest)		1.27	
**Hukou#PCHC**			
Rural-Q1 (poorest)			0.68
Rural-Q2			1.08
Rural-Q3			1.11
Rural-Q4			1.13
Rural-Q5 (richest)			1.50***
**Hukou#Year#PCHC**			
Urban-2018-Q1 (poorest)			2.10***
Urban-2018-Q2			2.54***
Urban-2018-Q3			2.37**
Urban-2018-Q4			1.33*
Urban-2018-Q5 (richest)			1.52*
Rural-2018-Q1 (poorest)			2.62***
Rural-2018-Q2			2.18***
Rural-2018-Q3			1.68***
Rural-2018-Q4			1.49***
Rural-2018-Q5 (richest)			1.17
**Observations**	15,489	15,489	15,489

All models included individual and household level covariates and district fixed effect. Standard errors adjusted for clustering at district level. All expenditure results were converted to comparable expenditure in 2018 with Beijing’s CPI. ***, **, and * indicated the significance at 1%, 5%, and 10% level, respectively

For results of every model, odds ratio was reported

*PCHC* Per capita annual household consumption

## Discussion

The rural and urban residents’ social health insurance schemes were integrated in Beijing in 2017. The rural people have begun to enjoy the equal benefit package as the urban people since the January 1st, 2018, which is believed as a critical strategy for improving equity in health. Using two waves of household survey data in Beijing in 2013 and 2018, it found that utilization of outpatient care substantially increased for all people with faster growth rate for the urban residents, poor residents used less inpatient care compared with the rich residents but suffered more heavily catastrophic health expenditures in 2018. For making the health insurance integration strategy really meaningful in improving equity in health care utilization and financial protection, a more pro-poor health protection scheme is needed targeting the rural and low-income people.

Income and price are two determinants of healthcare utilization. Between 2013 to 2018, utilization of outpatient care increased about 100% and users’ expenditure on that increased about 50%. During this time, residents’ per capita disposable income increased about 50% in Beijing thus people could afford more for healthcare [32]. Although income elasticities of health spending were highly relied on the context of health systems and socioeconomic contexts, most empirical studies, to our knowledge, observed an income elasticity of health spending lower than 1.5 [37–41]. Thus, increase of income might only explain part of the substantial increase of outpatient care utilization and expenditure in this study. A decreasing cost-sharing would also stimulate the demand for healthcare [42–44]. Nevertheless, after insurance integration, the reimbursement rate for outpatient care was almost unchanged. Thus, reimbursement rate might only explain very small part of changes on outpatient care in our study.

For urban residents, reduction on deductibles might account for most of the increasing utilization of outpatient care. After integration, for urban residents, deductibles of outpatient care decreased from 650 RMB yuan to the same deductibles as NCMS: 100 RMB yuan in primary healthcare institutions and 550 RMB yuan in secondary and tertiary hospitals. For outpatients with spending under 650 RMB yuan, reduction on deductibles might decreased both their average and marginal costs of outpatient care; for outpatients with spending higher than 650 RMB yuan, it only decreased their average outpatient spending but didn’t change the marginal cost of outpatient care. Thus, the reduction of deductibles might attract more low-spending outpatients, veiling part of its stimulating effect on outpatients’ expenditure.

For rural residents, their utilization of outpatient care also increased a lot. The increase of their healthcare utilization might be associated with the promotion of their insurance pool. Before integration, NCMS in Beijing was pooled at district level. If NCMS enrollees in district A use healthcare in medical institutions in other districts, the reimbursement rate for the healthcare utilization would be much lower or even 0. Moreover, for NCMS enrollees, if they visited a medical institution only covered by URBMI, they would also get a much lower reimbursement or even no reimbursement from NCMS. After integration, URRBMI covers all medical institutions covered by NCMS and URBMI, leading to more health providers with normal reimbursement rate available, especially for the rural people. If more medical institution is available, it would also reduce patients’ indirect healthcare costs such as the time and traffic costs.

After the integration, the reimbursement rate for inpatient care increased by 5–10 percentage points for urban residents and over 30 percentage points for some rural residents. Even so, we observed little changes on the utilization of inpatient care. Relative to outpatients, inpatients always suffer more severe diseases and their utilization of inpatient care might be less responsive to price and convenience of healthcare [42, 45]. Another possible reason is that some hospitalization might be substituted with outpatient care. Improved access to outpatient care would reduce patients’ risk of hospitalization, especially for those with chronic conditions [46, 47]. In addition, utilization of inpatient care has rapidly increased in China, whose discharge rate has exceeded the average of OECD countries [48, 49]. The price effect on hospitalization is weakening.

The poorest 2 group rural residents’ utilization and expenditure increased relatively more. Compare to others, they might have more unmet healthcare demand and be more responsive to the change of reimbursement policy. Insurance integration substantially improved their reimbursement for hospitalization, stimulating their demand for inpatient care. In addition to the poorest rural residents, people who were already hospitalized were also more likely to respond to the change of reimbursement. In our study, only richer residents’ annual spending on hospitalization increased. It implied that capacity to pay still played an important role in residents’ responses to healthcare price.

For most rural residents and poor urban residents, most of their hospital admissions might be necessary. Although their utilization of outpatient care increased a lot, their demand for inpatient care didn’t decline. For the poorest 2 group, their use of inpatient care even increased in 2018. For the wealthy urban residents, however, our results implied that there were some room for the reduction of their utilization of inpatient care. Guiding the rich to use inpatient care in a more rational way might be a practical approach for promoting the efficiency and equity of health system.

Moreover, it should be noted that we just analyzed the association between insurance policy and residents’ out-of-pocket expenditure, which was expected to decrease with the reimbursement rate. Thus, although the increase of out-of-pocket expenditure was not significant, patients’ total spending on hospitalization might significantly increase, especially for patients whose reimbursement rate increased a lot after integration.

After the insurance integration, both rural and urban residents’ benefit packages improved, but their risk of having catastrophic health expenditure, especially poor residents’ risk, also increased. Disparities in utilization of outpatient care between residents with different household consumption narrowed. Similar consumption on outpatient care, however, meant heavier financial burden for poor residents. It reminded the policy makers that in the process of narrowing the inequity in healthcare utilization, the inequity in financial protection might widened if lacking efficient measures to control the financial burden from the additive healthcare utilization. To improve the health equity both in healthcare utilization and financial protection, more pro-poor insurance policies are needed. Moreover, the cost of hospitalization was high. In 2018, patients’ annual spending on inpatient care, was almost half of rural residents’ per capita annual expenditure (or more than 1/5 of urban residents’ per capita annual expenditure) [32]. Utilization of inpatient care was highly associated with individuals’ capacity to pay. Thus, pro-poor policies are also important for improving equity in inpatient care utilization.

CHE in our study was about 20% in 2018, which was a relatively higher estimation than some other studies using data of CHARLS or CFPS [10, 11]. When using NHSS data, we estimated the annual spending on outpatient care and self-treatment by multiplying two-week spending by 26, which would overestimate the spending of who reported any utilization of outpatient care or self-treatment and thus overestimate their risk of suffering CHE. In our study, we focused more on the changes of some key outcomes instead of the values of these outcomes themselves. The systematic overestimation of CHE might have limited impact on our key results. Two recent studies using NHSS data of Jiangsu province showed a higher incidence of CHE, about 30%, even they calculated CHE at household level and with a threshold of 40% [12, 13].

Beijing is one of the most developed cities in China and its urban area is also more developed than the urban areas of other cities. Thus, our results might display a higher healthcare utilization and lower CHE than analysis of other cities. The gap of development between Beijing and other cities make it possible for other cities’ policy makers to learn from Beijing’s practice and to prepare more for the upcoming changes or challenges their society may face after further development.

There are several policy implications for China and other developing countries. First, pro-poor policies are important in a unified insurance system. As mentioned above, although the reimbursement rate for hospitalization was higher than outpatient care, the average out-of-pocket spending of inpatient care was much higher than outpatient care. Poor patients were more likely to deal with their disease by outpatient care. In the context of current health insurance system, social health insurance provides a relative lower financial protection for outpatient care. Thus, social health insurance plays a relatively limited role in reducing low-income residents’ financial risks due to their increasing access to healthcare (most of them are outpatient care). In addition, financial barrier to essential healthcare would make the poor risk more serious health problems and further influence their capacity to earn income. The pro-poor policies would improve poor residents’ capacity to pay not only by lowering the price of healthcare for the poor but also by secure their ability to earn.

Under limited budget, more pro-poor reimbursement policies should be at a higher priority for policy makers. One of the pro-poor policies is the income related copayment ceiling used by countries like Korea and Germany [20, 24]; another kind of policy is the differential reimbursement rate for different level of services, which is used by countries like Singapore [50]. For basic healthcare, which is highly needed by low-income residents, the reimbursement rate is high; for healthcare with more comfortable experience or unnecessary healthcare, the reimbursement rate is lower. For most developing countries, due to lacking adequate budget for healthcare and lacking good income recording system, differential reimbursement rates may be a more practical approach. Of course, it’s also of vital importance to monitor the implement of these insurance policies and adjust them according to the pattern of people’s healthcare utilization.

Second, some studies found that health insurance didn’t reduce residents’ out-of-pocket expenditure or financial risks [4, 5, 7]. Low reimbursement rate, which limited the insurance’s function of financial protection, was believed as one of the reasons for this phenomenon. Our study found, even for a relatively highly developed city with better health security system like Beijing, its residents’ financial risk still significantly increased with the healthcare utilization. For other less developed regions or countries, it’s might be impossible to reduce financial burden just by improving the reimbursement rate. Improving the efficiency of health system and avoiding unnecessary healthcare expenditure are also critical for the reduction of financial burden. This implies that not only the expanded policies such as higher reimbursement rate and more available healthcare resources, but also the constraint policies like rigorous health technology assessments and controls on excessive medical treatment are needed to improve the health equity of health system [51].

There are several limitations of our study. First, our data only covers the initial period after insurance integration, the long-term effect of this policy might be different. Second, between 2013 and 2018, some other reforms in Beijing and changes of some sociodemographic factors might also influence healthcare utilization and medical spending. Although we tried to consider those related policies in the analysis, we are not able to explain all of their possible effects with the data in a complex health system. Third, as mentioned above, the methodology of calculating CHE in our study and the underestimation of PCHC when using self-reported data might overestimate the CHE, but we think it have limited impact on our key results.

## Conclusions

This study investigates changes in health care utilization and financial protections after the integration of rural and urban social health insurance schemes. From 2013 to 2018, both rural and urban residents’ utilization of outpatient care increased. Compared to rural residents, urban residents were more likely to use outpatient care and this urban–rural gap became widened in 2018. Relative to outpatient care, utilization of inpatient care changed little. The utilization of inpatient care was still highly associated with individuals’ capacity to pay. Although the health insurance reforms in Beijing played a role in improving residents’ utilization of healthcare, they failed to reduce residents’ risks of suffering catastrophic health expenditure, especially for those were not well off. Our study advocates more pro-poor insurance policies and more efforts on the efficiency of health system.

## Supplementary Information

**Additional file 1.**

## Data Availability

The data that support the findings of this study are available from Beijing Municipal Health Big Data and Policy Research Center but restrictions apply to the availability of these data, which were used under license for the current study, and so are not publicly available. Data are however available from corresponding author upon reasonable request and with permission of Beijing Municipal Health Big Data and Policy Research Center.

## Abbreviations

NCMS: The rural new cooperative medical scheme
URBMI: Urban resident basic medical insurance scheme
URRBMI: Urban and rural resident basic medical insurance scheme
CHE: Catastrophic health expenditure
PCHC: Per capita annual household consumption
