# Supplementary material for: Changes in health care utilization and financial protection after integration of the rural and urban social health insurance schemes in Beijing, China
Source: BMC Health Serv Res. 2022 Oct 3;22:1226. doi: 10.1186/s12913-022-08602-1 (PMC9528155; doi:10.1186/s12913-022-08602-1)
Supplement: Supplementary file 1 — Additional file 1. [file 12913_2022_8602_MOESM1_ESM.docx]

**Supplementary file**

Since Chinese households are considered to intend to help members economically, we further develop another calculation of CHE to try to take this economical help into consideration.

$$E_{h}=1 if \frac{\sum_{i=1}^{i} OOP_{ih}}{i*PCHNFC_{h}}\geq0.25$$

$$E_{h}=0 if \frac{\sum_{i=1}^{i} OOP_{ih}}{i*PCHNFC_{h}}<0.25$$

$$CHE_{h}=\frac{1}{h}\sum_{h=1}^{h} E_{h}$$

$E_{h}$ was measuring whether household $h$ suffering CHE, $\sum_{i=1}^{i} OOP_{ih}$ is the sum of out-of-pocket health expenditure of $i$ household members with NCMS, URBMI or URRBMI in household $h$, $PCHNFC_{h}$ is the per capita household non-food consumption of household $h$, $CHE_{h}$ is the incidence of CHE. We analyzed the change of financial protection using this household CHE and Table S1 at the end of this response file shows the results. As the results shows, our analysis using individual CHE was robust.

Table S1 Regression results for incidence of individual CHE and household CHE.

|  | **Model 1** | | **Model 2** | | **Model 3** | |
| --- | --- | --- | --- | --- | --- | --- |
|  | **Ind. ^a^** | **Fam. ^b^** | **Ind. ^a^** | **Fam. ^b^** | **Ind. ^a^** | **Fam. ^b^** |
| **Rural** | 1.08 | 1.07 | 1.01 | 1.10 |  |  |
| **PCHC (poorest as the reference)** | | | | | | |
| Q2 | 0.81*** | 0.80*** | 0.84 | 0.76** | 0.62*** | 0.65 |
| Q3 | 0.73*** | 0.74*** | 0.85 | 0.91 | 0.62* | 0.73 |
| Q4 | 0.74*** | 0.67*** | 1.00 | 0.92 | 0.72 | 0.86 |
| Q5 (richest) | 0.72*** | 0.61*** | 1.02 | 1.07 | 0.60* | 0.77 |
| **Hukou#Year** | | | | | | |
| Urban-2018 | 1.96*** | 1.70** |  |  |  |  |
| Rural-2018 | 1.78*** | 1.86*** |  |  |  |  |
| **Year#PCHC** | | | | | | |
| 2018-Q1 (poorest) |  |  | 2.42*** | 2.56*** |  |  |
| 2018-Q2 |  |  | 2.29*** | 2.76*** |  |  |
| 2018-Q3 |  |  | 1.88*** | 1.81*** |  |  |
| 2018-Q4 |  |  | 1.46*** | 1.51** |  |  |
| 2018-Q5 (richest) |  |  | 1.27 | 0.95 |  |  |
| **Hukou#PCHC** | | | | | | |
| Rural-Q1 (poorest) |  |  |  |  | 0.68 | 0.84 |
| Rural-Q2 |  |  |  |  | 1.08 | 1.05 |
| Rural-Q3 |  |  |  |  | 1.11 | 1.16 |
| Rural-Q4 |  |  |  |  | 1.13 | 0.92 |
| Rural-Q5 (richest) |  |  |  |  | 1.50*** | 1.36** |
| **Hukou#Year#PCHC** | | | | | | |
| Urban-2018-Q1 (poorest) |  |  |  |  | 2.10*** | 1.95*** |
| Urban-2018-Q2 |  |  |  |  | 2.54*** | 2.55** |
| Urban-2018-Q3 |  |  |  |  | 2.37** | 2.20** |
| Urban-2018-Q4 |  |  |  |  | 1.33* | 1.03 |
| Urban-2018-Q5 (richest) |  |  |  |  | 1.52* | 1.15 |
| Rural-2018-Q1 (poorest) |  |  |  |  | 2.62*** | 2.87*** |
| Rural-2018-Q2 |  |  |  |  | 2.18*** | 2.87*** |
| Rural-2018-Q3 |  |  |  |  | 1.68*** | 1.65*** |
| Rural-2018-Q4 |  |  |  |  | 1.49*** | 1.75** |
| Rural-2018-Q5 (richest) |  |  |  |  | 1.17 | 0.87 |
| **Observations** | 15,489 | 8,418 | 15,489 | 8,418 | 15,489 | 8,418 |

^a^ Incidence of catastrophic health expenditure at individual level.

^b^ Incidence of catastrophic health expenditure at household level.

PCHC, per capita annual household consumption.

All models included individual and household level covariates and district fixed effect. Standard errors adjusted for clustering at district level. All expenditure results were converted to comparable expenditure in 2018 with Beijing’s CPI. ***, **, and * indicated the significance at 1%, 5%, and 10% level, respectively.

For results of every model, odds ratio was reported.
